# Supplementary material for: Proof-of-Concept for Liquid Biopsy Disease Monitoring of MYC-Amplified Group 3 Medulloblastoma by Droplet Digital PCR
Source: Cancers (Basel). 2023 Apr 28;15(9):2525. doi: 10.3390/cancers15092525 (PMC10177279; doi:10.3390/cancers15092525)
Supplement: Supplementary file 1 [file cancers-15-02525-s001.zip › cancers-2292983-supplementary.pdf]

**Table S1.** patient characteristics, including age at diagnosis, sex, histology, WHO grade and information on methylation analysis.

| Patient Number | Age at diagnosis (years) | Sex | WHO Grade | Histology | Methylation Array | Methylation group (subgroup) | Methylation score | version of classifier |
|---|---|---|---|---|---|---|---|---|
| 1 | 11 | female | 4 | CMB | Illumina 450k | nonWNT/nonSHH; (II) | 0.60 | Medulloblastom classifier[23] |
| 2 | 9 | female | 4 | LCA | Illumina EPIC | nonWNT/nonSHH; (III) | 0.94 | Medulloblastom classifier[23] |
| 3 | 1 | male | 4 | CMB | Illumina 450k | nonWNT/nonSHH; (II) | 0.99 | Medulloblastom classifier[23] |
| 4 | 4 | female | 4 | anaplastic MB | Illumina EPIC | nonWNT/nonSHH; (II) | 0.58 | Medulloblastom classifier[23] |
| 5 | 8 | male | 4 | CMB | Illumina EPIC | nonWNT/nonSHH; (II) | 0.66 | Medulloblastom classifier[23] |

**Table S2.** Methods overview.

| Methods overview | | |
|---|---|---|
| **Number of CSF samples** | | 49 |
| | intraoperative | 1 |
| | external ventricular drain | 2 |
| | Ommaya reservoir | 43 |
| | lumbar puncture | 3 |
| **Number of blood samples** | | 9 |
| **Sample volume** | blood | 1-3ml |
| | CSF | 0.5-1ml |
| **Processing after collection** | CSF | |
| | | immediate freezing at -20°C |
| | | transfer to -80°C |
| | | centrifugation at 1.0G for 10min |
| | blood | |
| | | immediate centrifugation at 1.4G for 10min |
| | | storage of plasma/serum at -20°C |
| | | transfer to -80°C |
| | | centrifugation at 1.0G for 10min |
| **Storage vials** | various collection tubes | |
| **Isolation kit** | tissue | ReliaPrep gDNA Tissue Miniprep System |
| | blood | Quick-cfDNA/RNA serum & plasma kit; (Zymo Research Corp., Irvine, CA, USA) |
| | CSF | Quick-cfDNA/RNA serum & plasma kit; (Zymo Research Corp., Irvine, CA, USA) |

**Table S3.** Sample overview.

| Sample overview | Patient 1 | Patient 2 | Patient 3 | Patient 4 | Patient 5 |
|---|---|---|---|---|---|
| **CSF samples** | 14 | 10 | 11 | 5 | 9 |
| **Blood samples** | 3 | 2 | 1 | 1 | 2 |
| **Age of samples at analysis** | 7 | 4 | 8 | 5 | 0 |
| **Sampling sites** | | | | | |
| intraoperative | | 1 | | | |
| EVD | | | | | 2 |
| lumbar puncture | 2 | | | | 1 |
| Ommaya reservoir | 12 | 9 | 11 | 5 | 8 |
| **Median AR (CSF)** | 9.776 | 3.171 | 34.83 | 1.55 | 25.88 |
| **Mean AR (CSF)** | 11.97 | 3.574 | 42.77 | 2.534 | 396.9 |
| **% of positive ddPCR analyses (CSF)** | 100% | 90% | 100% | 20% | 100% |
| **% of positive cytology results** | 5.4% | 16.8% | 16.3% | 17.3% | 11.4% |

Figure S1A *MYC* region on Chromosome 8 from the primary tumor of patient 1. Estimated log2 copy number ratio (CNR): 0.6; Scatter plot of ddPCR analysis of tumor tissue from patient 1: green droplets are positive for *MYC*, blue droplets positive for reference gene (AP3B1), orange droplets positive for both (*MYC* & *AP3B1)*

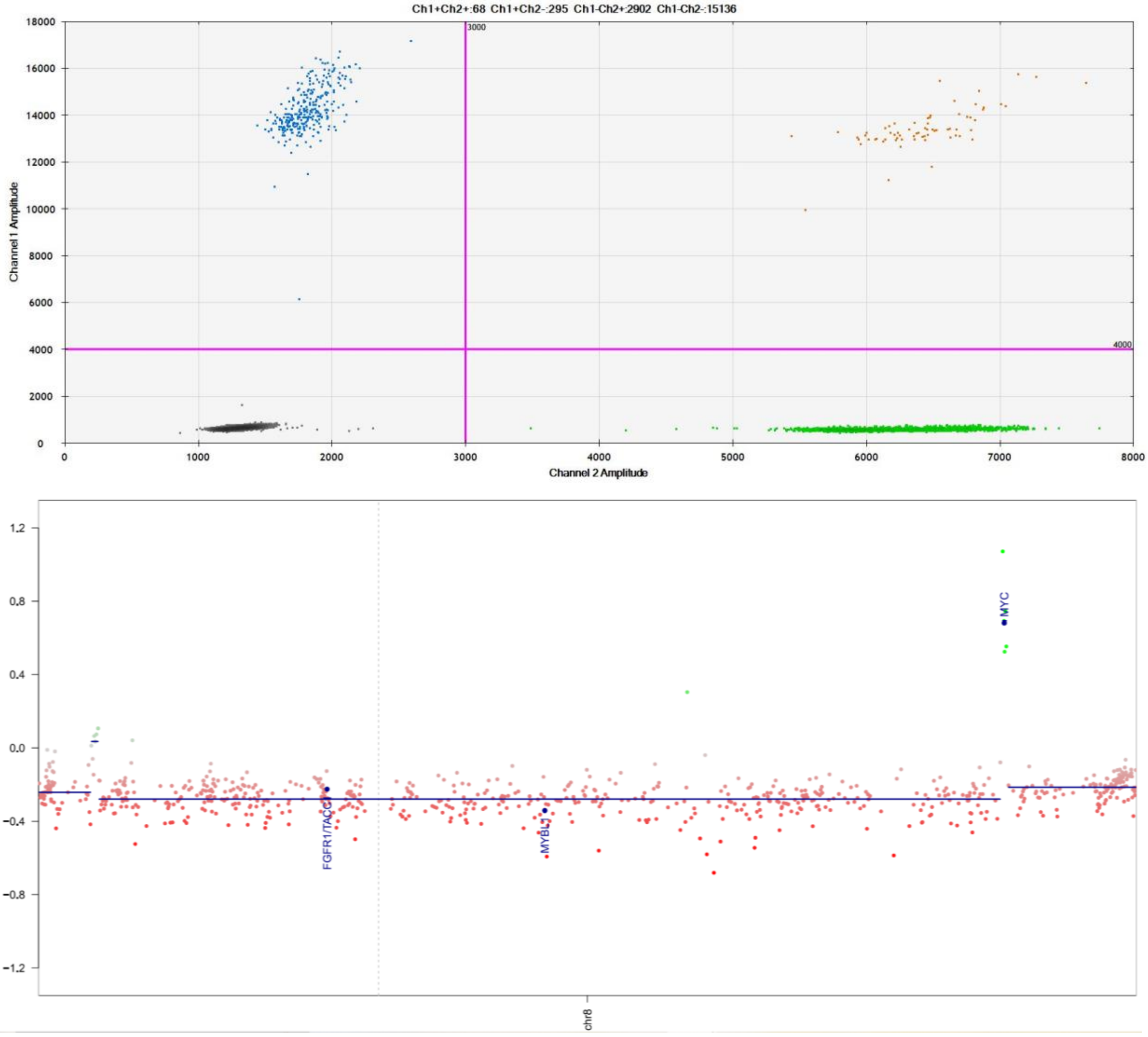

Figure S1B: *MYC* region on Chromosome 8 from the primary tumor of patient 2. Estimated log2 copy number ratio (CNR): 0.6;

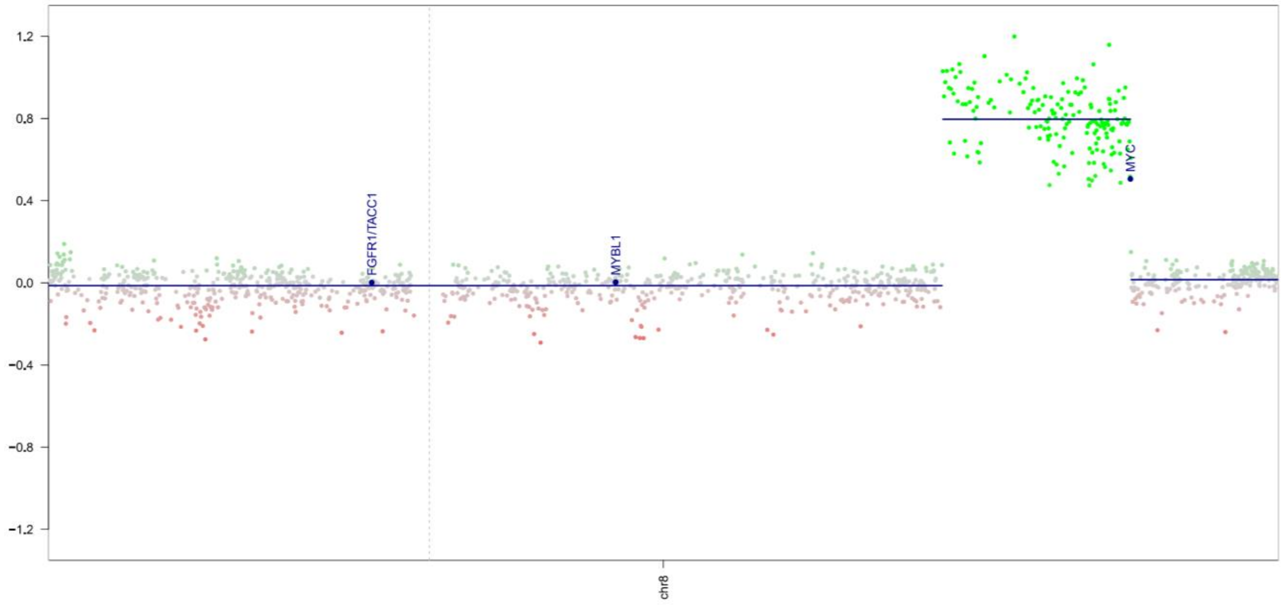

Figure S1C: *MYC* region on Chromosome 8 from the primary tumor of patient 3. Estimated log2 copy number ratio (CNR): 1.1; Scatter plot of ddPCR analysis of tumor tissue from patient 3: green droplets are positive for *MYC*, blue droplets positive for reference gene (AP3B1), orange droplets positive for both (*MYC* & *AP3B1)*

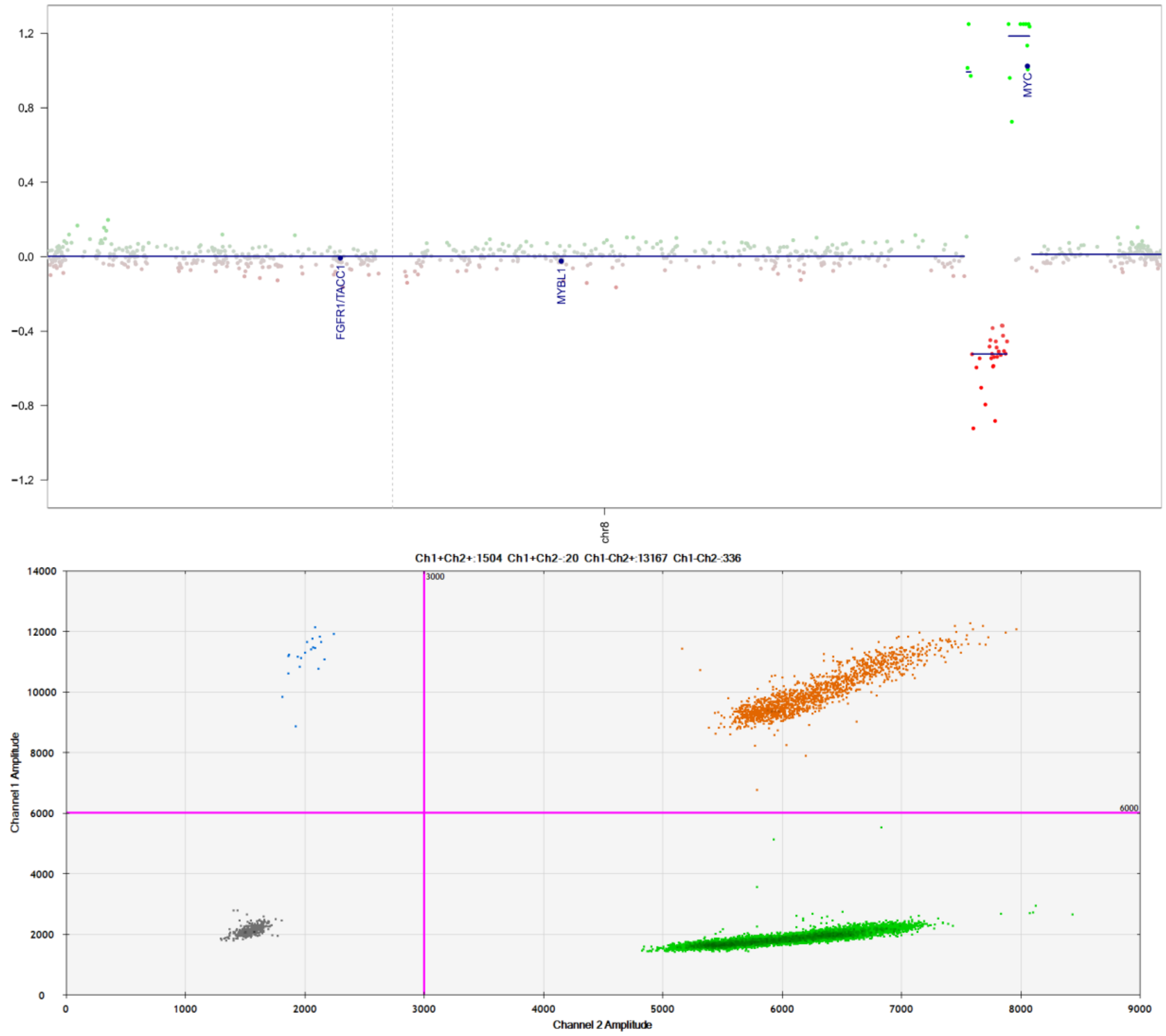

Figure S1D: *MYC* region on Chromosome 8 from one metastasis of patient 4. Estimated log2 copy number ratio (CNR): 0.3; Scatter plot of ddPCR analysis of matching tumor tissue from patient 4: green droplets are positive for *MYC*, blue droplets positive for reference gene (AP3B1), orange droplets positive for both (*MYC* & *AP3B1)*

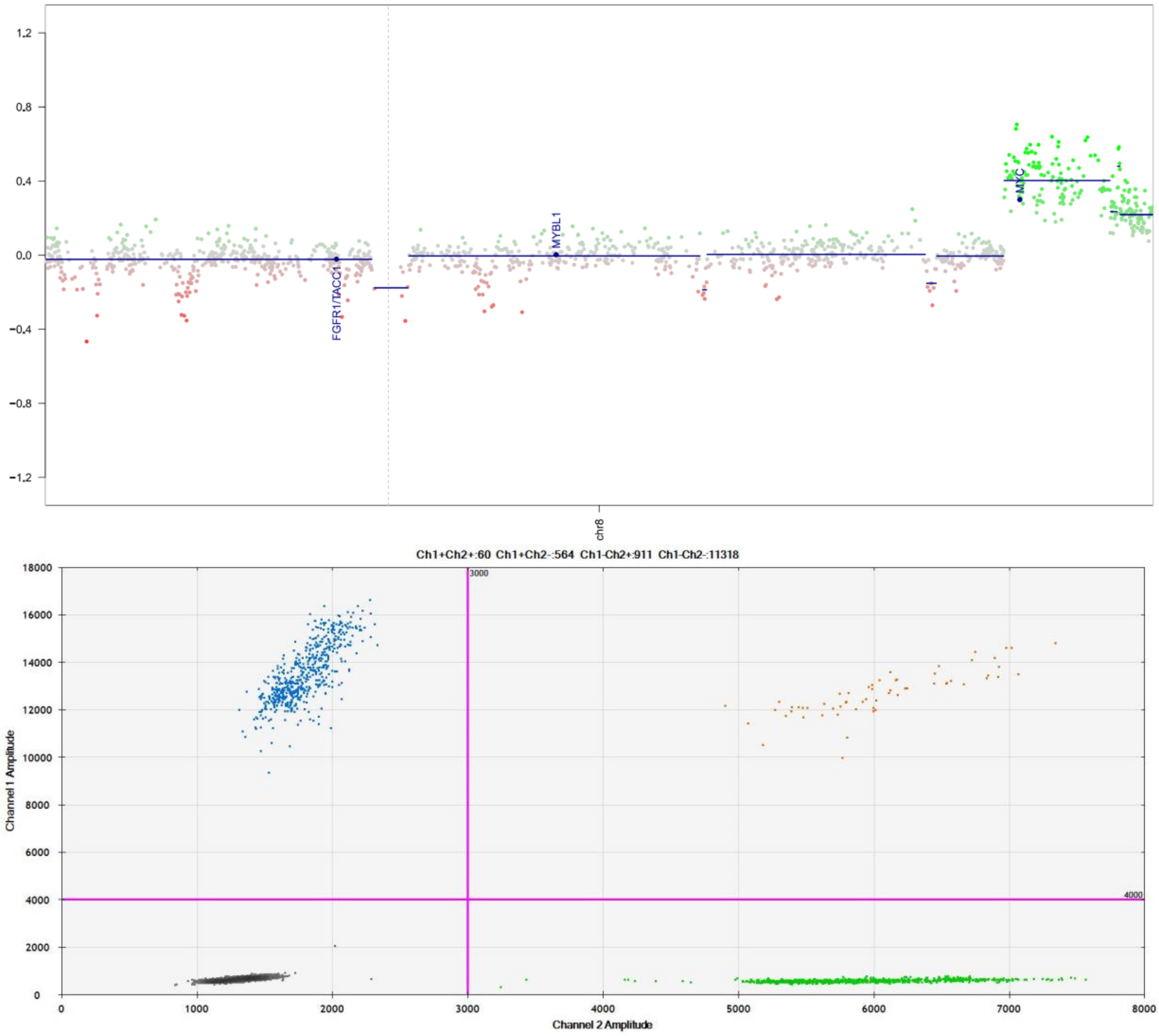

Figure S1E: *MYC* region on Chromosome 8 from the primary tumor of patient 5. Estimated log2 copy number ratio (CNR): 0.6; Scatter plot of ddPCR analysis of tumor tissue from patient 4: green droplets are positive for *MYC*, blue droplets positive for reference gene (AP3B1), orange droplets positive for both (*MYC* & *AP3B1*

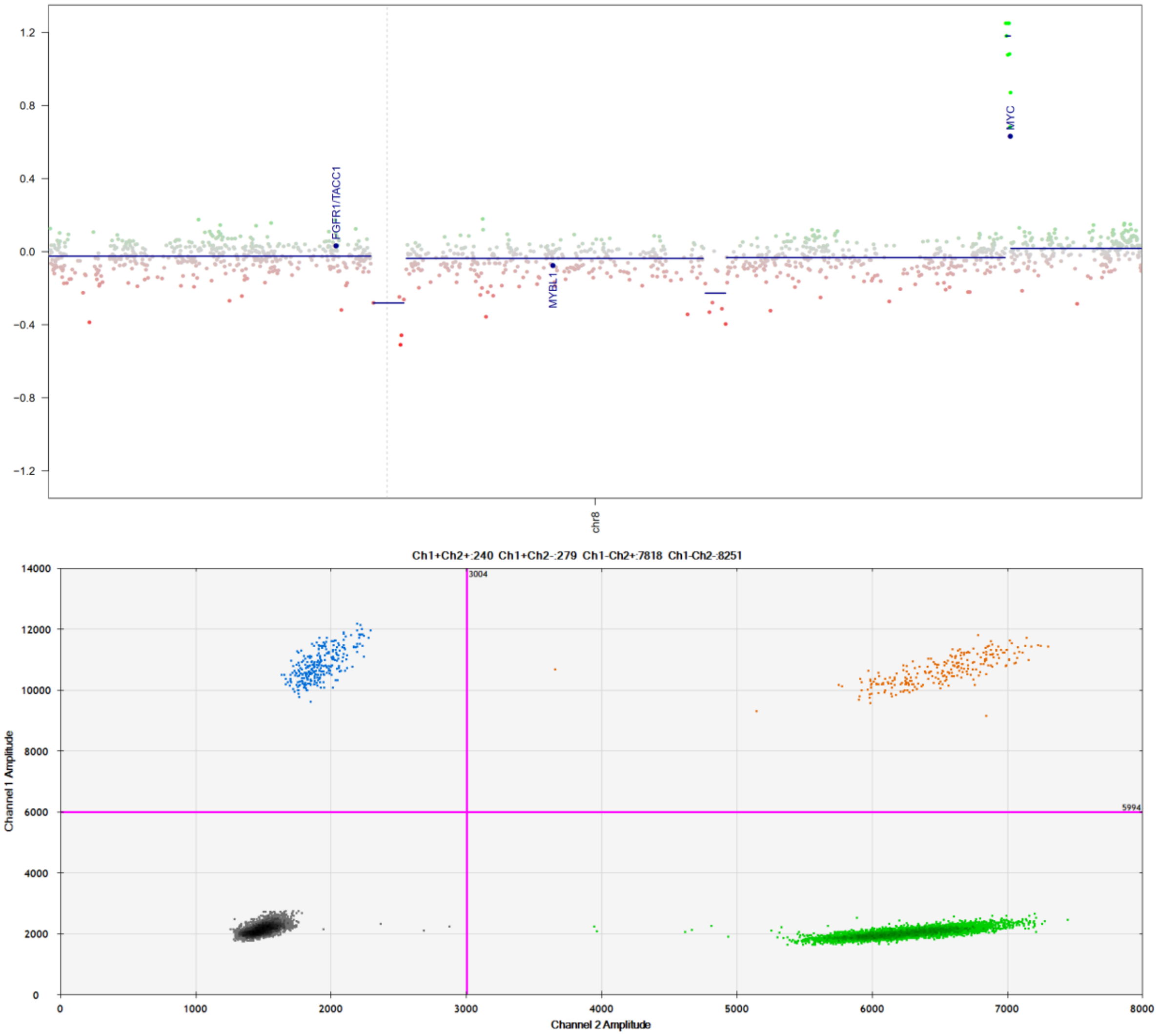

Figure S2: Violin Plot showing the amplification rate of CSF samples from *MYC* amplified MB patients compared with controls. Controls included three patients with non-*MYC* MB, including Group 3/4 with MYC polysomy (n=1), one patient with MB group 3/4, one patient with MB SHH, as well as samples from patinets with ependymoma (n=1), low grade glioma (n=1), craniopharyngioma (n=1) and a patient with a suspected pituitary apoplexy without malignancy (n=1)

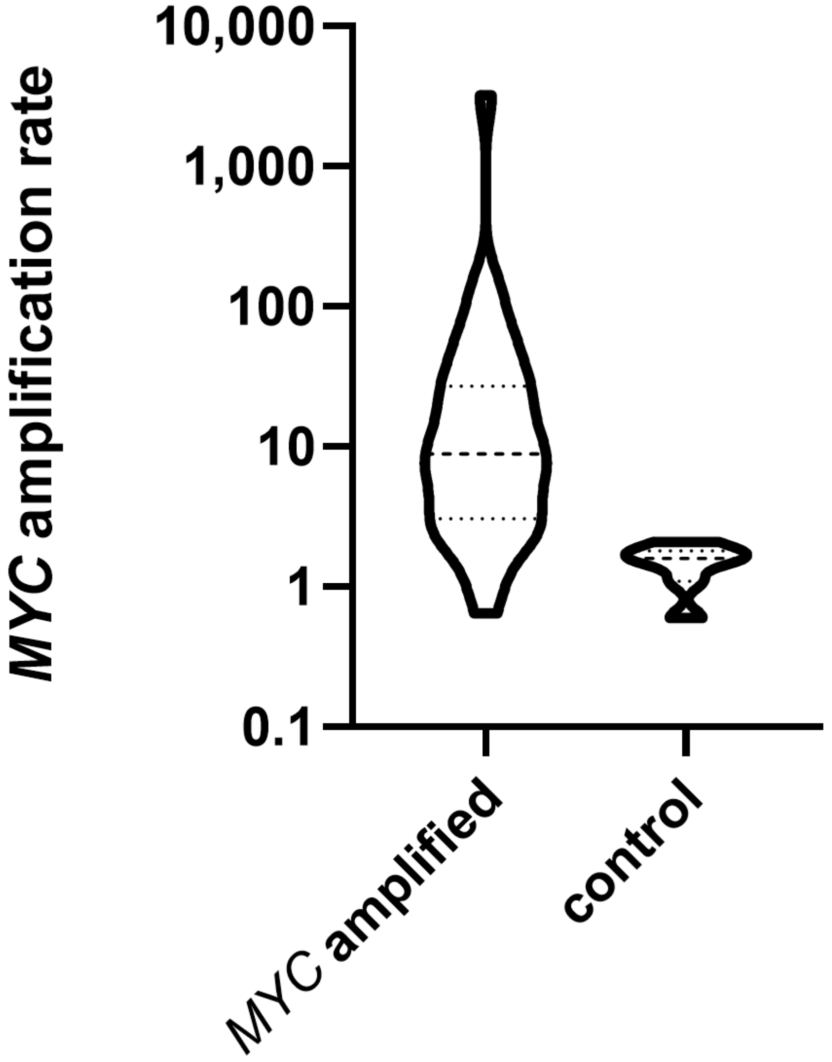

Figure S3: *MYC* amplification rate by ddPCR in the CSF of two patients (1&3) aquired on five consecutive days during one treatment week with etoposide administered intraventricularly via an Ommaya reservoir.

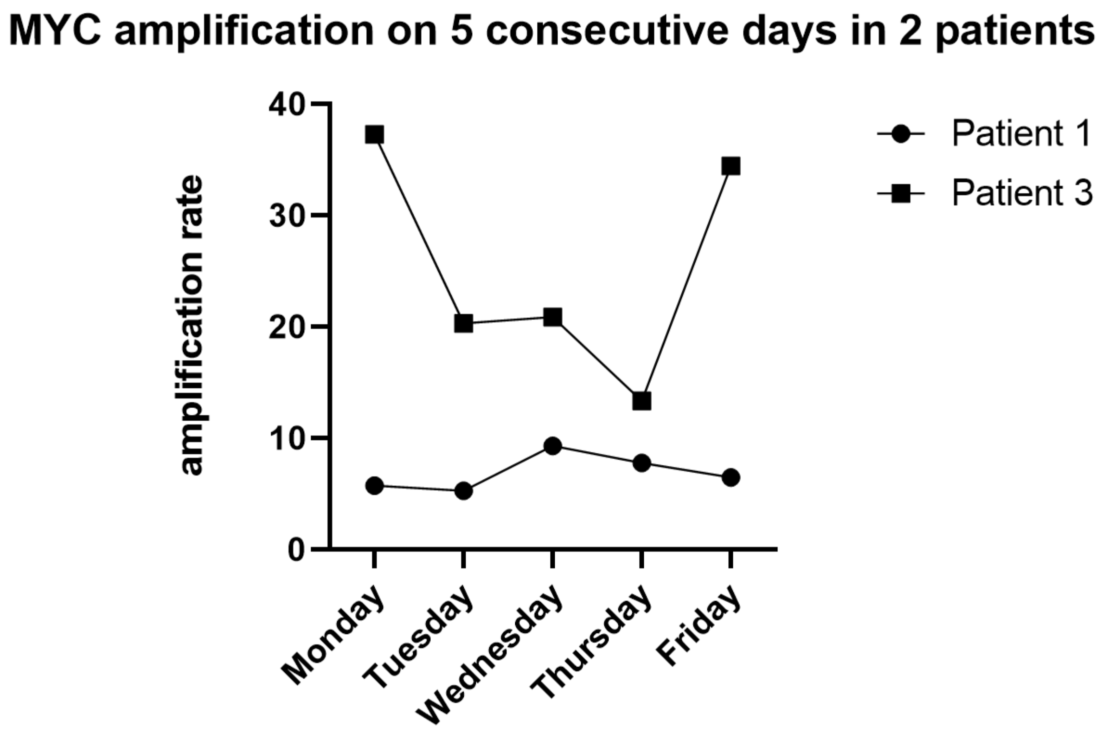

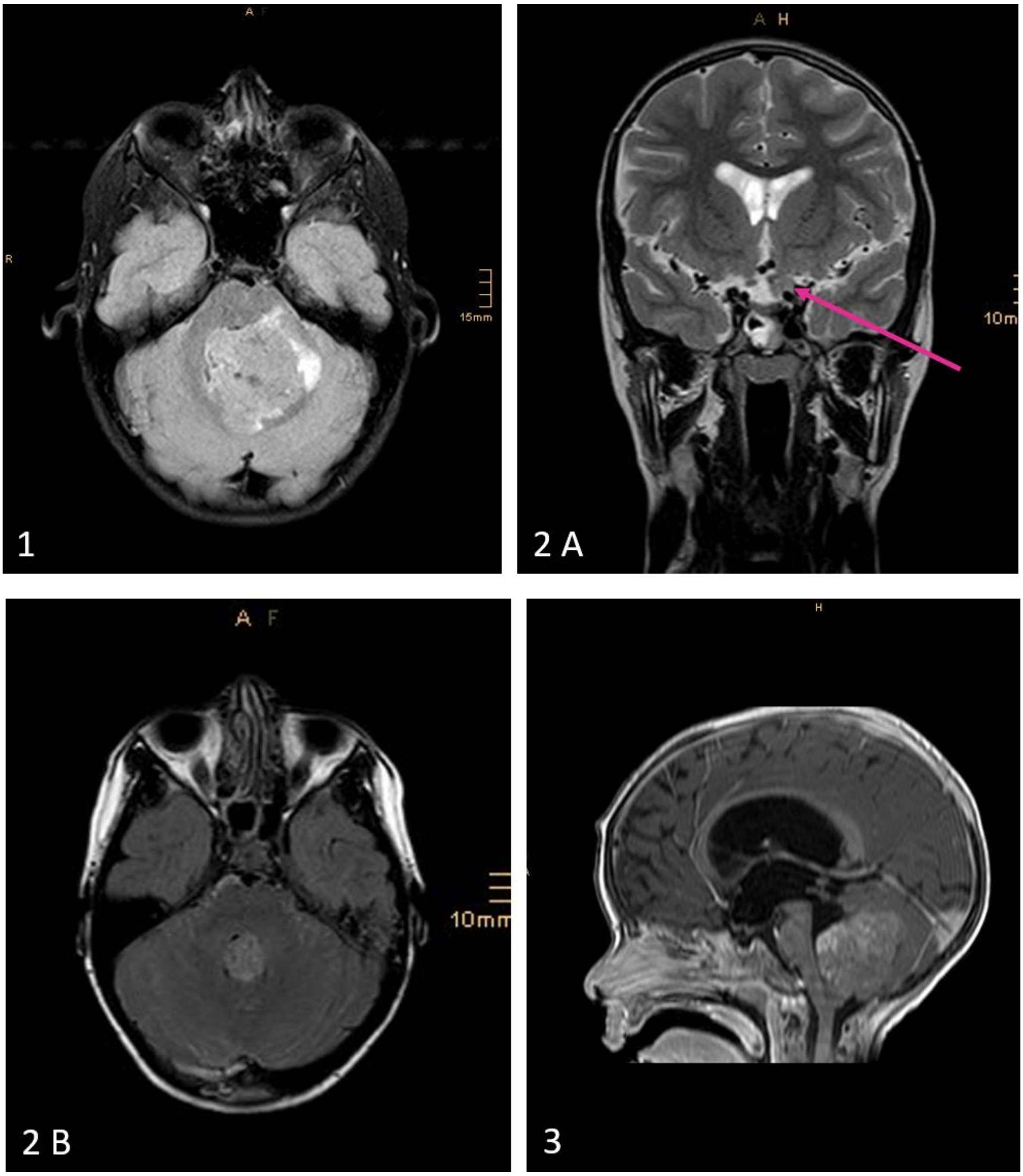
A F
R
15mm
1
A H
2 A
A F
10mm
2 B
H
3

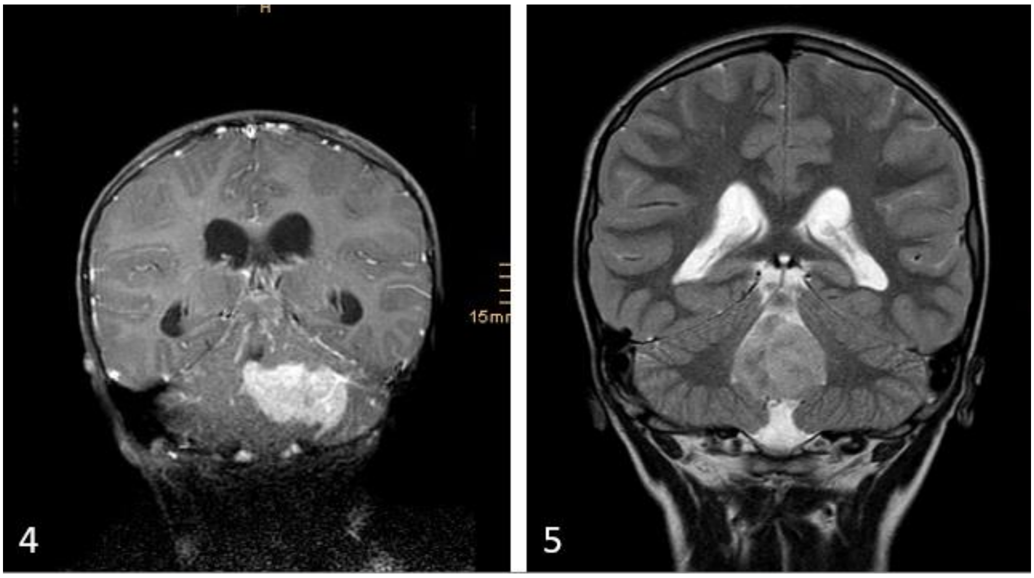


Figure S4: Representative MRI images at diagnosis from each patient 1-5.

1:  Transversal FLAIR (Fluid attenuated inversion recovery) image showing large slightly hyperintense mass located in the 4th ventricle;

2: A: Coronal T2-weighted image representing a mass adjacent to the left optical nerve (arrow) with incipient nerve dislocation & B: transversal FLAIR image 3 weeks later showing an additional hyperintense mass of the vermis;

3: Sagittal T1-weigted contrast-enhanced image depicting a large mass located in the 4th ventricle with moderate contrast enhancement and consecutive hydrocephalus;

4: Coronal T1-weighed contrast-enhanced image showing a vividly enhancing mass of the left cerebellar hemisphere;

5: Coronal T2-weighed image with a heterogeneous predominantly moderate hyperintense large mass located in the 4th ventricle.
